# Systematic Review on the Mental Health and Treatment Impacts of COVID-19 on Neurocognitive Disorders

**DOI:** 10.3390/jpm11080746

**Published:** 2021-07-29

**Authors:** Laura Dellazizzo, Nayla Léveillé, Clara Landry, Alexandre Dumais

**Affiliations:** 1 Research Center of the Institut Universitaire en Santé Mentale de Montréal, 7331 Hochelaga, Montreal, QC H1N 3V2, Canada; laura.dellazizzo@umontreal.ca (L.D.); nayla.leveille@umontreal.ca (N.L.); clara.landry@umontreal.ca (C.L.); 2 Faculty of Medicine, Université de Montréal, 2900 Edouard Montpetit Blvd, Montreal, QC H3T 1J4, Canada; 3 Institut National de Psychiatrie Légale Philippe-Pinel, 10905 Blvd Henri-Bourassa E, Montreal, QC H1C 1H1, Canada

**Keywords:** neurocognitive disorders, mental health, treatment, COVID-19, systematic review

## Abstract

**Objectives.** The COVID-19 pandemic has had many public health impacts, especially on vulnerable individuals including adults with neurocognitive disorders (NCD). With increasing literature, this systematic literature review aimed to address the mental health effects of COVID-19 on people with NCD in addition to examine the impact of the pandemic on treatments/resources for NCD. **Methods.** A literature search was conducted in the electronic databases of PubMed, PsycINFO, Web of Science and Google Scholar. Studies were included so long as they assessed the mental health or therapeutic effects of COVID-19 on NCD. **Results.** Among the retrieved articles, 59 met eligibility criteria. First, the pandemic and resulting self-isolation led to many detrimental effects on psychological well-being. Exacerbation and relapses of neurocognitive and behavioral symptoms were observed, as well as emergences of new psychological symptoms (i.e., depression, anxiety). Second, therapeutic and community services for individuals suffering from NCD, such as social support services and outpatient clinics, were disrupted or reduced leading to postponed appointments and evaluations, as well as reduced access to medications. These issues were somewhat palliated with the growth of telemedicine. **Conclusions.** This systematic review highlights the extent of the effects of the pandemic, and the topics addressed should be taken into consideration by healthcare practitioners, institutions, and policymakers to ensure that proper measures are employed to protect this population from additional harm.

## 1. Introduction

COVID-19 has infected over 115 million people and caused over 3 million deaths worldwide [1]. Although most people contracting the virus experience mild/moderate symptoms that dissipate without treatment, more vulnerable individuals (e.g., elderly populations with health comorbidities, psychiatric populations) may develop severe complications that can lead to hospitalization, intubation, and/or death [1]. To slow down the propagation of the virus, strict confinement measures and limited social contacts have had to be deployed [2]. Although contexts differ substantially, a parallel may be drawn between this situation and solitary confinement in correctional settings that consist of placing inmates in restricted housing with increased security for a prolonged amount of time, often for up to 23 hours per day, with strictly limited social contacts. A systematic review and meta-analysis on the latter matter has indeed established moderate associations between placement into solitary confinement and mental health deteriorations [3]. Coincidentally, home confinement during the pandemic has likewise imposed a burden on the physical and psychological well-being of the general population and of more vulnerable individuals with pre-existing mental health issues as evidenced by several reviews [4,5,6,7]. Overall, the pandemic has brought forth an overall worsening of psychiatric symptoms and greater psychological distress in individuals, particularly the elderly, with mental health needs [8,9].

Notably, the World Health Organization has given interest to older adults with cognitive impairments and/or neurocognitive disorders (NCD) and has indicated that they are more susceptible to experiment adverse mental health effects caused by the pandemic [10]. NCD are challenging diseases associated with the alteration of one’s cognitive and behavioral abilities in a way that disrupts the person’s daily activities [11,12]. Common signs and symptoms include memory deficits, language problems, personality changes, agitation, anxiety, and depressive symptoms [11,12], which have been shown to be exacerbated by the pandemic [13,14,15]. People with NCD require a multifaceted approach, including but not limited to, medication, cognitive interventions, environmental measures, and exercise interventions [11,12]. Nevertheless, in such times where they might need more medical support than ever, COVID-19 has resulted in many canceled/postponed medical appointments [14] that could be detrimental to their health, as well as the emergence of telemedicine, though the latter’s efficacy for these populations remains a debated topic [2,16,17]. In all, such changing circumstances and growing research on the topic call for a thorough analysis via systematic review of the effects of the pandemic on the symptoms and quality of care of people with NCD, whose mental health needs are often overlooked. This systematic review discusses two main topics that have emerged from literature: (i) The effects of COVID-19 on psychiatric symptoms, and (ii) the effects of COVID-19 on treatments and resources for individuals with NCD.

## 2. Methodology

### 2.1. Search Strategy

A search was independently conducted by a graduate student (L.D.) and a medical student (N.L.) in the electronic databases of PubMed, PsycINFO, and Web of Science (from databases’ inception date to December 2020). The search string focused on keywords in titles and abstracts. Search terms were chosen to be inclusive COVID-19 (e.g., “coronavirus”, “severe acute respiratory syndrome coronavirus 2”, “COVID”) and NCD (e.g., “dementia”, “Alzheimer’s disease”, “cognitive impairment”). The search syntax was tailored for each database. A secondary search was then conducted in Google Scholar to retrieve grey literature, and reference lists of included manuscripts were screened to ensure at best possible that no pertinent studies were missed. No setting, date or geographical restrictions were applied. Searches were limited to English and French language sources.

### 2.2. Study Eligibility

To maximise the number of studies and obtain an overall view of the subject, all study designs, including editorials, that evaluated the mental health effects of COVID-19 on patients with NCD were eligible. The inclusion of a study was based on: (i) The evaluation of the effects of COVID-19 on mental health in patients with NCD; and (ii) the assessment of change in the delivery of mental healthcare for patients with NCD. To ensure consensus, discussions on the inclusion of studies were held with the research team. Studies were excluded if they (i) did not provide a clear definition of their sample or the sample was not particularly on NCD patients (e.g., individuals with Parkinson’s disease, seniors with subjective cognitive decline, nursing home residents), (ii) evaluated the general physical health effects of COVID-19; (ii) were on the impact of COVID-19 in patients affected with the virus; (iii) aimed at evaluating the effects for caregivers or healthcare workers; and (iv) comprised of posters, preprints, non-peer reviewed reports, study protocols with no available data, non-accessible manuscripts, or papers from the media.

### 2.3. Data Extraction

Key information related to the study design, the sample characteristics and the outcome measured (mental health effects or therapeutic changes) were independently extracted form by L.D. and N.L. To achieve a high standard of reporting data, the *Preferred Reporting Items for Systematic Reviews and Meta-Analyses* (PRISMA) guidelines was followed [18].

## 3. Results

**Figure 1:**
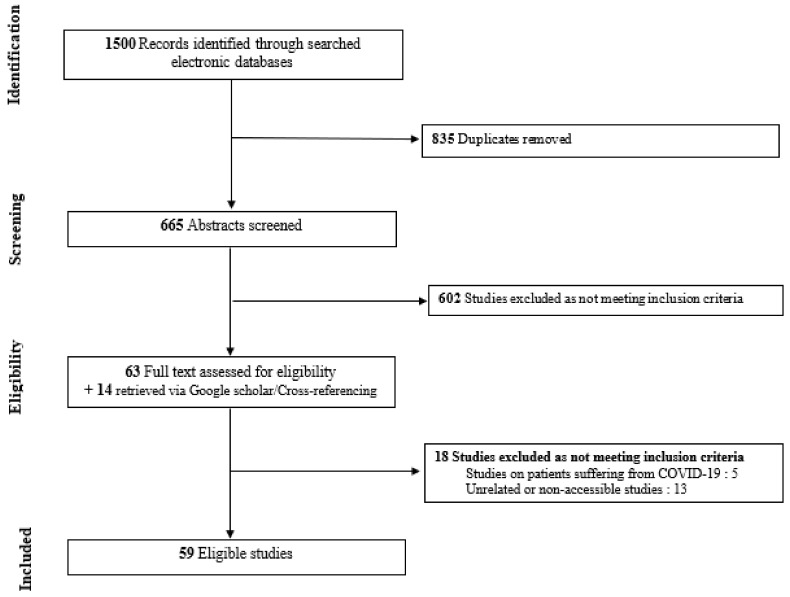
Flow-chart depicting the search strategy employed to find the studies included in the review.

Literature search identified 665 articles that were screened for eligibility after removing duplicates. Among the articles retrieved, 59 met our eligibility criteria. The PRISMA flowchart for the inclusion of studies in the review is shown in Figure 1. They were then categorized according to their relation to either (i) the mental health effects based on DSM-5 [19] symptomatology (k = 34:14 editorials/commentaries, 2 case reports, 9 cross-sectional/survey-based studies, 6 longitudinal studies, 3 reviews) or (ii) the therapeutic effects (k = 35:18 editorials/commentaries, 7 cross-sectional/survey-based studies, 5 longitudinal studies, 5 reviews) of the COVID-19 pandemic on individuals with NCD. A few studies pertaining to both these objectives were included in both categories. Concerning diagnoses provided by the included data-driven articles pertaining to mental health effects, 10 studies were conducted on people living with dementia (PLWD) in general, 2 were specifically conducted on individuals with Alzheimer’s disease (AD) and 4 included patients suffering from dementia or mild cognitive impairment (MCI). As for evidence-based literature provided from data-driven studies pertaining to therapeutic effects, 11 studies were conducted on PLWD in general, 2 were specifically conducted on individuals with AD, 1 was conducted on individuals solely suffering from MCI and 3 included patients suffering from dementia or MCI.

**Figure 2:**
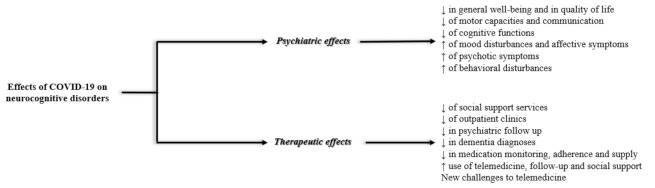
Summary of the findings provided from the systematic review.

See Figure 2 for a summary of highlights and Supplementary Material (Tables S1 and S2) for details on the retrieved papers. Within the sections below, we begin with evidence-based literature provided from data-driven studies (e.g., longitudinal studies, cross-sectional/survey-based studies) and end with highlights from non-data-driven studies (e.g., editorials) when available.

### 3.1. Psychiatric Effects

The pandemic and resulting self-isolation have led to many changes in relation to the psychological well-being of individuals suffering from NCD. Exacerbation and relapses of neurocognitive and behavioral symptoms have been observed, as well as emergences of new psychological symptoms.

Regarding cross-sectional data, several articles discussed an overall increase in both psychological and behavioral symptoms. A first cross-sectional study conducted on 204 caregivers of older adults with MCI or dementia found a significant overall decline, with communication, mood, movement, and compliance to new measures being most affected based on a self-reported questionnaire referring to any changes observed during the pandemic in physical, psychological and routine activities [13]. Another cross-sectional study conducted by telephone survey on 139 PLWD showed that 54.7% of them experienced worsened or emerging behavioral disturbances with agitation, aggression, apathy, and depression [14]. Results from a survey cohort on 121 symptomatic patients with NCD showed that almost half of patients reported an increase of one or more psychological symptoms (e.g., loneliness, anxiety, uncertainty and depression) and over 30% experienced social isolation [20]. Increases in behavioral symptoms, such as apathy, changes in sleeping behavior and aggression were also noted. Similar findings were observed in an editorial with cross-sectional findings conducted by the retrospective review of the electronic records of 634 patients who attended clinic consultations between 23 January and 1 June 2020, with 444 records of patients having visited the clinic before confinement and 190 records of patients having visited the clinic during confinement. Results showed a significant increase in the proportion of PLWD presenting behavioral changes, mainly agitation, sleep disturbance and irritability, during the period with home confinement restrictions compared to prior confinement (*p* < 0.001). Moreover, patients were more likely to exhibit exacerbations of behavioral and psychological symptoms in dementia (BPSD) scores (*p* = 0.002) [15]. A cross-sectional study by Penteado et al. [21] found increases in mood symptoms, sleep problems, and psychotic disturbances. More specifically, their elderly group comprising of 71 individuals primarily with mild or major NCD showed increases in anxiety (65%), feeling of insecurity (44%), discouragement (36%) and irritability (35%). In a multicenter nationwide cross-sectional survey by Cagnin et al. [22] having obtained via a structured telephone interview 4913 caregiver views on neuropsychiatric symptoms 1 month after the pandemic’s restrictions, there was a worsening of neuropsychiatric symptoms in over half of PLWD. Effects more particularly related to dementia type have shown that AD was associated with anxiety and depression (OR = 1.35, CI = 1.12–1.62), dementia with Lewy bodies (DLB) with worsening hallucinations (OR = 5.29, CI = 3.66–7.64) and sleep disorder (OR = 1.69, CI = 1.25–2.29), frontotemporal dementia (FTD) with wandering (OR = 1.62, CI = 1.12–2.35) and change of appetite (OR = 1.52, CI = 1.03–2.25). Cohen et al. [23] also noted that when comparing the frequency of behavioral symptoms within each dementia group category after the first 8 weeks of quarantine, anxiety, depression, and insomnia were more prevalent in subjects with mild dementia in comparison to those with severe dementia. This was based on a survey conducted on 119 Argentinian caregivers of persons with AD or related dementia living at home.

Concerning more particularly affective symptoms, a cross-sectional study on 39 caregivers of patients with AD or related dementia reported a 48% increased anxiety in the context of the lockdown reported by a questionnaire survey including questions about the level of anxiety before the pandemic and during the pandemic [24]. Of these 39 patients, 7 increased benzodiazepine doses since the beginning of the pandemic. Another cross-sectional study conducted on 126 outpatients with AD found that patients with more severe forms of cognitive impairments had substantially lower scores on the Geriatric Depression Scale (GDS) than those suffering from mild cognitive impairment when measured at the end of the first lockdown [25]. These findings were explained by the fact that patients with more severe forms of NCD did not fully understand the context and the seriousness of the outbreak. As for cognitive symptoms, a cross-sectional study conducted by telephone survey including standardized questions about the perceived changes in the patient’s clinical conditions that occurred in the last 30 days during confinement on 139 PLWD showed an overall worsening of cognitive symptoms, particularly memory and orientation abilities in one third of the sample. A functional decline was noticed in 19 patients and was mainly described in terms of dependence in personal care and housekeeping [14]. According to caregivers’ reports in the cross-sectional study by Penteado et al. [21], 34 elders showed changes in cognitive status, suggesting cognitive decline. An increase in psychotic symptoms may have likewise led to an increase in the need of medications for patients with NCD during the pandemic. For instance, a cross-sectional study on 80 family caregivers of persons with AD or related dementia described 12 patients needing an increase in antipsychotic doses during the lockdown reported by these caregivers in a questionnaire survey including questions about changes in medications since the pandemic [24]. This was also stated in a correspondence [26].

These cross-sectional findings have been supported by several longitudinal studies as well. One conducted on 36 PLWD from the *Brain and Body* program via phone call assessments with patients’ caregivers carried out 7 months before confinement (baseline assessment) and 3 months after home confinement (follow-up assessment) showed that care recipients significantly declined their independence in activities of daily living (*p* = 0.003) and increased their Neuropsychiatric Inventory scores (NIS) measuring delusions, agitation, and motor disturbances (*p* = 0.015). Moreover, 80% of caregivers mentioned that care recipients presented cognitive declines and 44.4% worsened in their behavioral and psychological symptoms [27]. Increases in NIS from before to 5 weeks after lockdown (*p* = 0.028) were also observed by another longitudinal study on MCI (*n* = 20) or mild AD (*n* = 20) [28]. The most affected neuropsychiatric symptoms were apathy and anxiety in patients with mild cognitive impairments, and apathy, agitation and aberrant motor behavior in patients with AD. Yet, no differences in quality of life, nor hallucination/delusion severity were noted. A third longitudinal study on 32 PLWD comparing patients’ last assessment before COVID-19 to a telemedicine assessment during the pandemic showed a significant worsening since last visits mostly in behavior (56%), language (47%) and cognitive functions (53%). Memory was described as being worse in 17 out of 32 patients [29]. A fourth longitudinal study on 38 patients with AD assessed by a clinician before and after the beginning of the pandemic depicted a slightly different impact of the pandemic on patients’ psychological well-being [30]. Only 10 presented neuropsychiatric changes during confinement and they had had lower general cognitive functioning at the time of their last Memory Clinic in comparison to those who did not show such changes. Based on on-site caregivers assessing depression and anxiety in participants with mild AD who live in retirement homes, participants reported higher depression (*p* = 0.005) and anxiety (*p* = 0.004) during than before the pandemic [31]. These increases were stipulated by authors to be attributed to the isolation of the residents and/or to the drastic changes in their daily life and care they receive. Lastly, in a prospective study by Barguilla et al. [32] on 60 MCI and dementia patients from DegMar registry evaluating neuropsychiatric changes (assessed with the NPI) compared to previous follow-up within 6 months before the pandemic, authors noted that neuropsychiatric profiles globally worsened (*p* < 0.001), mainly in terms of agitation (*p* = 0.003), depression (*p* < 0.001), anxiety (*p* < 0.001) and appetite changes (*p* = 0.004). Moreover, 60% of patients had cognitive worsening as reported by caregivers and 15% presented delirium episodes.

The impacts of the pandemic on the psychological symptoms and well-being of PLWD were also discussed in non-systematic reviews [33,34,35]. Mok et al. [33] observed that the home-confinement of people with NCD for extended periods was commonly associated with frustration or other behavioral symptoms. Preoccupation with negative news of the pandemic may also have triggered negative emotions. In addition, these effects may have been exacerbated by many day-to-day activities (i.e., outdoor exercise, social engagement, recreational rehabilitation programs) that were suspended/reduced [33]. Simonetti et al. [34] also provided evidence showing that apathy, anxiety and agitation were most frequently reported during the pandemic and appear to arise from social restrictions. Manca et al. [35] likewise noted the emergence and worsening of neuropsychiatric symptoms in older adults with dementia.

In a similar vein, a relapse and exacerbation in symptoms of NCD with increased fear, loneliness, apathy, and affective, cognitive as well as psychotic symptoms, difficulty to conform with imposed measures, a lack of understanding of the situation, reduction in quality of life and negative impacts of face masks on psychological well-being were reported in 13 editorials/commentaries and 2 case reports [2,15,36,37,38,39,40,41,42,43,44,45,46,47,48]. Noteworthy, the isolation and social distancing measures might have contributed to generate feelings of loneliness and abandonment, potentially triggering behavioral modifications in people with NCD [41,42]. Additionally, it has been stated that those with NCD can have sensorial deficits and perception troubles, including visual difficulties and the inability to recognize faces and emotions. Face masks and physical distancing can disrupt facial familiarity and make it more difficult to recognize emotional facial expressions, which can provoke distress [36].

### 3.2. Therapeutic Effects

As well as directly causing an impact on the psychological well-being of patients with NCD, COVID-19 has led to many disruptions in the services, therapies and treatments received by these patients. It has likewise led to a major increase in the use of telemedicine for the management and treatment of these disorders, showing both promising results and challenges.

#### 3.2.1. Challenges to Standard Care and Social Support Services

Since the lockdown, there has been a significant reduction in social support service usage, as observed by a cross-sectional study on 42 caregivers of PLWD and 8 PLWD conducted by a telephone interview in the aim of conducting qualitative analyses [49]. Another cross-sectional study on 569 participants, including 61 PLWD, 219 current caregivers, 66 former caregivers and 223 older adults conducted by an online survey, showed that weekly social support service usage and access to various services were significantly reduced with COVID-19. Higher variations in social support service hours significantly predicted increased levels of anxiety in people with NCD [50]. Similar observations were reported by an additional survey where over 30% of symptomatic patients were unable to go to day care or community care services [20]. Cohen et al. [23] likewise observed that there was a high rate of discontinuation of rehabilitation during the epidemic with 76% of elderly patients with dementia having discontinued physical therapy, 91% occupational therapy, and 77% cognitive rehabilitation.

These findings were corroborated by a prospective study conducted by Barguilla et al. [32] on 60 MCI and dementia patients from DegMar registry comparing data to previous follow-up within 6 months before the pandemic. Hence, 16% reported difficulties accessing medical care, 33% received medical phone assistance, 20% needed emergency care and 21% had changes in psychopharmacological therapies. Similarly, a registry based longitudinal study by Spalletta et al. [51] comparing cancellation rates for first or follow-up appointments for memory services during the pandemic compared to previous year found that over 60% of patients with mild or major NCD missed their first and follow-up appointments to memory service during the pandemic due to restrictions. Moreover, an interrupted time series study by Chen et al. [52] assessing the medium-term impact of the pandemic on referrals to secondary care mental health services revealed no post-lockdown longer-term acceleration rate for referrals of people with dementia.

Not only were there reductions in the number of consultations, referrals, and admissions, but a cross-sectional study noted that there was a reduction in the diagnosis of incident dementia (−39%) in April and May 2020 compared to 2019 [53].

A review evaluating the challenges of NCD care during the pandemic noted that outpatient clinics for PLWD were suspended to attempt to reduce transmission or to redeploy staff to work at an infectious ward. While usual medications would be continued, changes or deterioration in clinical status requiring adjustment in medications, or other interventions, may have been overlooked. Scheduled blood taking or neuroimaging appointments may have likewise been neglected [33]. Another comprehensive review by Simonetti et al. [34] showed that there was a surge in the dosage of medications to treat patients, including antipsychotics and mood stabilizers. Moreover, although there was increasing use of remote technology as a compensatory strategy to counterbalance the lack of non-pharmacological interventions, the evidence behind these approaches have been mixed.

The challenges associated with psychiatric follow-up during the pandemic, such as postponed appointments, a decrease in the review of medications, a disruption of support services for patients and the need for the use of telemedicine or remote care, were addressed by 6 editorials/commentaries [2,39,40,42,54,55]. Noteworthy, several different problems with medication administration and adherence may have arisen due to changes in patient’s routine (affecting medicine-taking behavior), reduced caregiver input if the caregiver must self-isolate, or reduced contact with their general practitioner or community pharmacist [2]. As primary care providers and specialists were being redeployed to address medical emergencies, these physicians were not available to work up NCD, which likely impacts diagnosis and clinical management. Some hypotheses about the impact of the pandemic on medication supply for patients with NCD were also discussed by editorials. Patients who were stable on medications may have been impacted if the supply of their medication was disrupted due to missed visits, disruption of pharmacy pickup or delivery, or supply chain problems during the pandemic. Initiating a new medication during the pandemic may have been associated with higher risk, particularly if components of routine screening were disrupted. A limitation on resources and a need for physical distancing suspended non-pharmacological interventions (e.g., pet therapy, social groups) and resulted in increased isolation, a lack of physical exercise, decreased social engagement, and a suspension of purposeful activity [39,42].

#### 3.2.2. Increase in Telemedicine and Challenges

Regarding these new challenges that have risen since the pandemic and lockdown, telemedicine has been regarded as a possible avenue for NCD care. Telemedicine has successfully been implemented to partly palliate these service disruptions in social support, psychiatric or therapeutic consultations, group therapies, clinical assessment, etc.

Indeed, a cross-sectional study conducted via a telephone survey on 47 community-dwelling older adults living with MCI or mild dementia part of the TV-AssistDem clinical trial, receiving a TV based assistive integrated technology and treatment, compared to 46 patients receiving treatment as usual, showed no significant differences in health and well-being [56]. Another cross-sectional study conducted by a non-profit organization for patients with cognitive impairment studied the impact of switching appointments and consultations from in person to online by estimating the average number of visits before the state of alarm declaration and comparing these estimates with the observed number of weekly visits afterwards [57]. After one week, a drop of 60% in consultations was observed, but within 6 weeks, the foundation had returned to 78% of their regular activities, which showed capabilities to adapt to the crisis with telemedicine.

These findings were further reinforced by 2 longitudinal studies. A first longitudinal study on 32 PLWD receiving center care compared patients’ telemedicine assessments to the last in-person assessment at the clinic. Most were consistently satisfied with telemedicine visits and expressed their willingness to continue the telemedicine program in a satisfaction survey conducted after a few days following the beginning of telemedicine consultations [29]. A second longitudinal study comparing the impact of additional services delivered to both care-recipient and caregiver through video conference (*n* = 30) was compared with telehealth targeted at caregivers of community-dwelling people with cognitive impairment by telephone alone (*n* = 30) [58]. The intervention with supplementary telehealth delivered via videoconferencing was associated with resilience against a decline in general cognitive functioning over 4 weeks in a pretest-posttest design, with interviews being conducted at baseline and follow-up.

Many reviews also described the emergence of telemedicine since the pandemic for patients with NCD and its effects. More specifically, a non-systematic review evaluating the use of telemedicine in PLWD suggested that remote assessment was acceptable to patients and caregivers. Informed consent, informant history and attention to privacy and autonomy were primordial. Some neuropsychological tests administered by videoconferencing showed good agreement with in-person assessment, though they lacked validation and norms. Aspects of the remote NCD neurological examination have been performed reliably by telemedicine [59]. A systematic review including 12 articles assessed the use of telemedicine in rural areas for PLWD since the beginning of the pandemic and observed mixed results. Overall, there was general satisfaction with telemedicine (for patients, caregivers, referring physicians and healthcare professionals). However, adherence and cognitive tests reported mixed results regarding the reliability of telemedicine, which may partly be attributed to the lack of accessibility to telemedicine in such setting [60].

The use of telemedicine and its benefits, including reduced outpatient visits in crowded hospital, minimized travel time to clinics, reduced waiting lists and similar efficacy to face-to-face meetings or activities, were also discussed in 6 editorials [16,41,61,62,63,64]. Noteworthy, Cognitive Stimulation Therapy (CST), which is an evidence-based psychosocial group treatment for people with a diagnosis of mild to moderate NCD, has been adapted virtually since the pandemic. An editorial and case report discussed the transition, describing ten in-person CST groups who were successfully transitioned to virtual CST [61]. Moreover, experience from practitioners have suggested the feasibility of connecting people with NCD virtually using information communication technology, without concerns of confusion or disengagement. There even appears to be additional benefits with the novelty and sense of empowerment associated with the use of virtual technologies in patients [62,63].

Whereas telemedicine appears as a promising avenue, several limitations have nevertheless been described by 2 commentaries [2,40] and 4 editorials [16,37,64,65]. These include the lack, of availability of appropriate conditions to perform tele-consultations (quality of connection, patients’ ownership of webcam), knowledge and familiarity and caregivers with these modern technologies, and ethical concerns about patients’ confidentiality, which require caution and specific privacy policies [16]. As mentioned in a policy form [65], it is important to consider vulnerable and ethnic minorities suffering from NCD, who often receive inadequate care, and will now be exposed to new constraints because of the pandemic. Furthermore, virtual modalities may not be adequate to perform physical and neurological examinations or some of the cognitive tests required when diagnosing NCD or monitoring their progression [2]. Not all screening instruments used to assess cognitive function may be appropriate to be administered remotely, which may have affected assessment of some cognitive impairments [40]. Lack of access to technology, digital illiteracy, and sensory impairment limit the use of online resources [64]. Particularly, the use of telemedicine may be more difficult for patients suffering from LBD. These patients have significantly more visual and language impairments when compared to people with other NCD (e.g., difficulty reading, double vision, speech freezing, hypophonia), which may lead to struggles to virtually interact [37]. To palliate to the in-person service disruptions regarding psychiatric assessment, follow-up and social support, it will be essential to address these telemedicine challenges. Indeed, telehealth shows to be a promising avenue for patient care, even after the pandemic.

## 4. Discussion

COVID-19 has had many public health impacts especially on more vulnerable individuals, including older adults with NCD. Overall, although preliminary, this systematic review showed (i) a significant deterioration in the symptoms regarding several spheres (i.e., overall symptoms and well-being, cognitive symptoms, affective symptoms, behavioral symptoms and psychotic symptoms); and (ii) important therapeutic changes induced by the pandemic, as it brought challenges in standard care and social support services, which were mainly palliated with the growth of telemedicine.

Firstly, our review shed light on the extent of COVID-19′s effects on NCD symptoms. Both longitudinal and cross-sectional data described overall worsening and emergence of psychological and behavioral symptoms. For instance, an exacerbation of cognitive symptoms was observed in 80% of subjects [27]. The main spheres involved memory, orientation, motor abilities and language [29]. Further, a rise of affective symptoms (e.g., as anxiety and depression) [14,24] and behavioral symptoms (e.g., agitation, aggression, apathy and mood changes) were affected in 44.4% of subjects as established by a longitudinal study [27]. The quality of the evidence of these findings was at best low-to-moderate and was supported by longitudinal and cross-sectional literature, as well as by editorials and commentaries. An increase in psychotic symptoms was depicted, with a poor quality of evidence being based on limited case study and cross-sectional data [47,48]. Altogether, COVID-19 has caused a general functional decline in this population and has negatively impacted patients’ general well-being and quality of life [14,30,37]. This may be explained by numerous factors, including difficulty to understand the situation and to adhere to the imposed rules including face masks, which can cause distress in those with NCD by preventing them from recognizing faces and emotions [2,36,37,40,41,42,46]. Although elderly people with NCD, in both the community and long-term care homes, frequently rely on others to assist them in their daily activities, confinement restricted these contacts [66]. Moreover, community-dwelling older adults with NCD depend on various sociocultural activities and community services, especially if they live alone [39]. Those living in long-term care homes, who are generally at more advanced stages of their condition, were also deprived of essential socialization and cognitively stimulating activities due to strict confinement rules [39,67]. Hence, with increasing literature highlighting the negative effects of the pandemic on mental health and well-being in this vulnerable population, healthcare providers should be especially concerned and consider adapting their clinical approach to meet the specific needs on patients with NCD.

Besides, the observed effects may very well have been exacerbated by insufficient access to healthcare [2,12,14,42]. Notwithstanding, individuals with NCD depends on a variety of resources (e.g., community services, treatments) that help manage their condition, and maintain good quality of life [2,39,63]. However, our review highlighted that COVID-19 has caused a disruption and/or reduction of these resources [33,49,50], which could be explained by confinement measures aiming to limit social contacts by closing services and redeployment of medical staff from outpatient clinics to infectious wards [33]. Quality of evidence for these findings was relatively low-to-moderate based on some editorials, reviews and limited cross-sectional/longitudinal literature. As a result, the use of online therapeutic methods (e.g., online medical appointments, group cognitive stimulation therapy) has expanded significantly [2,61]. Promising results have not only been observed in NCD care, but in all medical fields [68]. Cross-sectional studies and reviews support that its efficacy could compare with that of in-person consultations [56,57,58,59]. Although telemedicine could bring additional benefits, such as feelings of pride [29,62,63], certain challenges need to be addressed, such as difficulties for these patients to adapt to technology, accessibility issues, and the adaptation of some interventions [2,64]. With these difficulties addressed, telemedicine could have major clinical implications and thus reform the care of patients with NCD.

To conclude, this systematic review addressed the health effects of COVID-19 on people with NCD, suggesting that it has worsened their symptoms and reduced/disrupted their care. It is worth mentioning that, as illustrated earlier, the latter plays an important role in the observed deterioration of symptoms, and thus both factors are intrinsically linked. Markedly, these issues have posed considerable challenges for caregivers of patients with NCD that should not be overseen [69,70]. The pandemic has also caused the rapid expansion in the use telemedicine. Nevertheless, some limitations of current literature need to be considered. Firstly, the populations included in represented considerable heterogeneity (i.e., diagnostic criteria for NCD, long-term care facilities vs community settings, caregiver vs patient assessment). Furthermore, small sample sizes rendered the results less generalizable. Secondly, the included articles and quality of evidence presented substantial heterogeneity. Unfortunately, a large proportion of studies was provided by editorials limiting the quality of evidence. However, findings tended to reach consistent conclusions. Although several cross-sectional and longitudinal studies have recently been emerging and could provide additional information, more high-quality longitudinal studies are needed to evaluate the true impact of the pandemic on those with NCD and its long-lasting sequelae. Lastly, this review was limited to English- and French-language, peer-reviewed literature published before December 2020. Although we employed a rigorous search strategy, it is possible that relevant literature was excluded in terms of language and timeframe. Relevant articles may have likewise been omitted due to the surge of literature focusing on COVID-19. Therefore, conclusions drawn from this review should be interpreted with caution and follow-up systematic reviews with higher quality data are warranted. It is worth noting that evidence provided in this review are in accordance with more recent findings being published on an ongoing basis (e.g., [71,72,73,74,75,76,77,78,79]). Despite these limitations, the current systematic review allowed to highlight the extent of the effects of the pandemic, and the results established should be taken into consideration by healthcare practitioners, institutions, and policymakers to ensure that proper measures are employed to protect this population from additional harm. For instance, further development of telehealth should be a priority in years to come to increase its accessibility [41]. Clinicians must adapt their practice to optimize remote delivery of care, while keeping in mind their patients’ best interest [41]. As for in-person consultations and social services, safety should be ensured with preventive measures, such as physical distancing, face masks and hand disinfection, while still providing a proper, personalized contact with this vulnerable population (e.g., clear face masks to facilitate communication and reduce anxiety) [36,49].

## Supplementary Materials

The following are available online at https://www.mdpi.com/article/10.3390/jpm11080746/s1, Table S1. Details of the retrieved studies in relation to the mental health impacts of COVID-19, Table S2. Details of the retrieved studies in relation to the treatment impacts of COVID-19.
