# Supplementary material for: Systematic Review on the Mental Health and Treatment Impacts of COVID-19 on Neurocognitive Disorders
Source: J Pers Med. 2021 Jul 29;11(8):746. doi: 10.3390/jpm11080746 (PMC8401453; doi:10.3390/jpm11080746)
Supplement: Supplementary file 1 [file jpm-11-00746-s001.zip › jpm-1283115-supplementary.pdf]

<u>Supplementary material</u>

Dellazizzo, L., Léveillé, N., Landry, C. & Dumais, A. Systematic review on the mental health and treatment impacts of COVID-19 on neurocognitive disorders.

Table S1. Details of the retrieved studies in relation to the mental health impacts of COVID-19

Table S2. Details of the retrieved studies in relation to the treatment impacts of COVID-19

Table S1. Details of the retrieved studies in relation to the mental health impacts of COVID-19

**Author, Year, Country**	**Study design**	**Methodology**<br>**i) Population**<br>**ii) Number of participants/groups**<br>**iii) Median/mean (SD) age**<br>**iv) Variables studied**	**Summary of results**
**Tsapanou, 2020 (13)**<br>**Grece**	Cross sectional	i) Caregivers of patients with MCI or dementia<br>ii) 204<br>iii) Caregivers : 59 (14), people with MCI/dementia : 79 (8.9)<br>iv) Self-reported questionnaire about any changes during the pandemic in physical, psychological and routine activities	• Overall decline : 78.8%
**Koh, 2020 (15)**<br>**Singapore**	Letter to the editor with cross sectional findings	i) Dementia patients who attended clinic consultations between January 23 and June 1st 2020, at the Centre of Geriatric Medicine, Tan Tock Seng Hospital, Singapore<br>ii) 634 : 444 having visited the clinic before and 190 having visited the clinic after confinement<br>iii) NA<br>iv) Retrospective review of the electronic records to evaluate behavior, stress and adjustment of medication	• Behavioral changes (37% vs. 23%; p<0.001)<br>• Increase in the level of stress reported by caregivers (22% vs. 9%; p<0.001)<br>• Need for adjustment of psychotropic medications (38% vs. 27%; p<0.001)
**Canevelli, 2020 (14)**<br>**Italy**	Cross sectional	i) Dementia patients attending the Center for Cognitive Disturbances and Dementia, Sapienza University of Rome<br>ii) 139 dementia patients and 43 patients with milder cognitive disturbances<br>iii) 79 (median)<br>iv) Standardized set of questions about perceived changes in their clinical conditions that occurred in the previous 30 days	• 31.7% : worsening of cognitive symptoms<br>• 19 patients : functional decline<br>• 54.7% : worsening/onset of behavioral disturbances
**Cohen, 2020 (24)**<br>**Argentina**	Cross sectional	i) Family caregivers of persons with AD or related dementia from the Aging and Memory Center at FLENI in Buenos Aires<br>ii) 80<br>iii) NS<br>iv) CDR anxiety score	• 43.8% : experienced anxiety before the pandemic without a statistical difference during lockdown<br>• 48% (39 patients) : increased level of anxiety after lockdown<br>- 12 needed an increase in antipsychotic doses

Author, Year, Country	Study design	Methodology<br>i) Population<br>ii) Number of participants/groups<br>iii) Median/mean (SD) age<br>iv) Variables studied	Summary of results
			• 7 needed an increase in benzodiazepine doses
**Tsugawa, 2020 (25) Japan**	Cross sectional	i) Outpatients with AD from the Memory Disorder Clinic at the Department of Geriatric Medicine, Tokyo Medical University<br>ii) 126 (51 mild AD, 75 moderate/severe AD)<br>iii) 80.98 ± 5.30 (mild), 83.27 ± 5.32 (moderate/ severe)<br>iv) Geriatric Depression Scale – Short version (GDS-S)	• Patients with more severe forms of cognitive impairments have substantially lower GDS scores than those suffering from mild cognitive impairment (2.54 ± 2.22 vs 5.73 ± 3.30, p < 0.0001)<br>• Mild AD : 81% patients aware of COVID-19, moderate/severe : 31%<br>• Mild AD : 78% patients understanding the reason for wearing a mask, moderate/severe : 24%
**Cagnin, 2020 (22) Italy**	Cross sectional	i) Patients with dementia and caregivers from 87 Centers for Cognitive Disorders and Dementia (CDCD) distributed among Northern, Center, and Southern Italy<br>ii) 4913 (AD = 3372, DLB = 360, FTD = 415, VD = 766)<br>iii) 78.3 ± 8.2<br>iv) BPSD : qualitative interview about worsening of symptoms 1 months after the introduction of pandemic restrictions	• BPSD changes in 59.6% patients (increase in BPSD : DLB = 63.8%, FTD = 55.3%, AD = 50.5%, VD = 50.3%)<br>• Modification of drug treatments in 27.6% of patients with BPSD changes<br>• AD : increase risk of anxiety and depression<br>• DLB : worsening of hallucinations and sleep disorder<br>• FTD : increase risk of wandering and change of appetite<br>• Increased stress in 65.9% caregivers
**Van Maurik, 2020 (20)**	Cross sectional	i) Patients with dementia, MCI and subjective cognitive decline (SCD) and their caregivers<br>ii) 389 (SCD = 268, MCI = 35, dementia = 86)<br>iii) 67 ± 8<br>iv) Survey on psychosocial effects of Corona measures (questions on COVID-19 infection, discontinuation of care, social isolation and psychosocial effects)	• 35% of symptomatic and 25% of cognitively normal patients : experience of social isolation<br>• Faster cognitive decline : worrisome for 53% caregivers, 44% of symptomatic patients and 14% of cognitively normal patients<br>• 56% caregivers : higher caregiver burden<br>• Increase in psychological symptoms : reported by 46% symptomatic patients,

**Author, Year, Country**	**Study design**	**Methodology**<br>**i) Population**<br>**ii) Number of participants/groups**<br>**iii) Median/mean (SD) age**<br>**iv) Variables studied**	**Summary of results**
			38% cognitively normal patients and 48% caregivers<br>• Increase in behavioral problems reported by 75% caregivers<br>• Social isolation (OR = 3.2)/ reporting of one or more psychological symptoms by the patient (OR = 8.1) : determinants for worries of faster cognitive decline
**Penteado, 2020** (21) **Brazil**	Cross sectional	i) Elderly patients with preexisting neuropsychiatric disorders and aging adults with DS<br>ii) 71 elderly patients and 29 DS patients<br>iii) Elderly : 76.8 ± 8.7, DS : 43.3 ± 13.4<br>iv) Informant's questionnaire : questionnaire to screen for the occurrence of psychiatric symptoms and changes in mental state, clinician's questionnaire : clinical aspects of the patient's mental state in the context of the pandemic, HADS : anxiety and depressive symptoms, NPI-Q : psychological/behavioral symptoms	• Increase in mood symptoms, sleep problems, and psychotic disturbances<br>• Increase in anxiety (65%), feeling of insecurity (44%), discouragement (36%) and irritability (35%) in the elderly group<br>• Patients with dementia : worst HADS score, NPI-Q scores and higher caregiver impact<br>• 34 elders : changes in cognitive status
**Cohen, 2020 (23) Argentina**	Cross sectional	i) Family members of patients at the Aging and Memory Center of FLENI with AD and related disorders living at home<br>ii) 119<br>iii) 81.16 ± 7.03 (patients)<br>i) Survey : onset/worsening of behavioral symptoms, change in prescription of psychotropics	• 60.5% subjects : new onset/exacerbation of behavioral symptoms (anxiety : 33%, depression : 12,8%, sleep disorders : 14.7%)<br>• Behavioral symptoms : associated with older age and presence of anxiety before the pandemic<br>• Overall increase in use of psychotropic medication<br>• Increased caregiver stress
**Borges-Machado, 2020 (27) Portugal**	Longitudinal	i) Dementia patients and caregivers from the *Brain and Body* project<br>ii) 36 caregivers of individuals with neurocognitive disorders (NCD)<br>iii) Caregivers : 64.94 ± 13.54, patients : 74.28 ± 6.76 years	• Significant decline in independence in ADL (p=0.003)<br>• Increased NPI total score (MD = 5.72; 95% CI: 1.19 to 10.25, p = 0.015)<br>• 80% cognitive decline

Author, Year, Country	Study design	Methodology<br>i) Population<br>ii) Number of participants/groups<br>iii) Median/mean (SD) age<br>iv) Variables studied	Summary of results
		iv) Pre and post confinement :<br>Care recipients' ability to function independently in ADL : Barthel Index (BI)<br>iv) BPSD : Neuropsychiatric Inventory (NPI)	• 44.4% worsened BPSD
**Capozzo, 2020 (29) Italy**	Longitudinal	i) Dementia patients receiving care at the Center for Neurodegenerative Disease and The Aging Brain of the University of Bari<br>ii) 32<br>iii) 66<br>Disease progression since last in person visit : Clinical Dementia Rating Scale–FrontoTemporal Dementia (CDR-FTD)	• Significant worsening since last visit in behavior (56%), language (47%), and cognitive functions (53%)<br>• Worsened memory in 17 out 32 patients
**Boutoleau-Bretonnière, 2020 (30) France**	longitudinal	i) AD patients who had a recent visit (two to four months prior to the study) to the Memory Center of the Hospital of Nantes-France<br>ii) 38<br>iii) 71.89 (8.24)<br>iv) Questions from NPIQ (specific neuropsychiatric symptoms and care givers' distress)	• 10 patients presented neuropsychiatric changes during confinement
**Lara, 2020 (28) Spain**	Longitudinal	i) Patients from the Cognitive Stimulation Program of the Cognitive Disorders Unit<br>ii) 40 (20 MCI, 20 mild AD)<br>iii) NS<br>iv) Evaluation before lockdown vs 5 weeks after lockdown :<br>Neuropsychiatric Inventory (NPI)<br>v) EuroQol-5D questionnaire (EQ-5D)	• Mean (SD) total baseline NPI score : 33.75 (22.28), compared with 39.05 (27.96) after confinement ($P = 0.028$)<br>• No differences in EQ-5D scores during the re-evaluation
**El-Haj, 2020 (31) France**	Longitudinal	i) Patients with AD living in retirement homes in France (MMSE >21/30 within 3 months before the study)<br>ii) 58<br>iii) Median = 71.79 years<br>Hospital Anxiety and Depression Scale rated by the patients for the period before the beginning of social distancing ad during social distancing	• Higher depression during COVID-19 crisis than before ($Z = -2.84$, $p = .005$, Cohen's $d = 0.80$)<br>• Higher anxiety during COVID-19 crisis than before ($Z = -2.86$, $p = .004$, Cohen's $d = 0.81$)<br>•
**Barguilla, 2020 (32)**	Longitudinal	i) Dementia and MCI patients from DegMar registry (Hospital del Mar)	• 70% : abandoned previous daily activities

Author, Year, Country	Study design	Methodology<br>i) Population<br>ii) Number of participants/groups<br>iii) Median/mean (SD) age<br>iv) Variables studied	Summary of results
**Spain**		ii) 60<br>iii) 75.4 ± 5.2<br>iv) Telephone questionnaire : "CogVid Hospital del Mar questionnaire" (functional and neuropsychiatric changes experienced by patients and caregivers) compared to previous follow-up within 6 months before the state of alarm	• 60% : cognitive worsening<br>• No significant change in functional status<br>• Overall increase of NPI score (3 to 8, p<0.000), with a significant difference in agitation, depression, anxiety and changes in appetite<br>• 15% : delirium episodes<br>41% caregivers : increased burden
**Mok, 2020 (33) China**	Review	i) Dementia patients<br>ii) NA<br>iii) NA<br>iv) NA	• Increased frustration/behavioral problems<br>• Negative emotions triggered by preoccupations with negative news of the pandemic<br>• Suspension of day-to-day activities exacerbating negative emotions
**Simonetti, 2020 (34) United States**	Comprehensive review	i) Dementia patients<br>ii) 20 articles included<br>iii) NA<br>iv) NA	• Increased hopelessness<br>• Increased apathy : most common manifestation from isolation due to Covid<br>• Increased anxiety and aggression, because of reduction of social contact<br>• Increased agitation
**Manca, 2020 (35) UK**	Review	i) Older adults with dementia<br>ii) 5 articles included<br>iii) NA<br>iv) NA	• Emergence/worsening of neuropsychiatric symptoms : increased agitation, apathy, depression, irritability<br>• Increase in NPI scores
**Padala, 2020 (47) USA**	Case report	i) Nursing home resident with Alzheimer's<br>ii) 1<br>iii) 81<br>iv) Evaluation of agitation and confusion	• Increasingly agitated and confused after the stop of his daughter's daily visits because of Covid
**El Haj, 2020 (48) France**	Case report	i) Patient with AD<br>ii) 1<br>iii) 84<br>iv) Hallucination Scale for 0 to 12	• Increase of 3 points from March 7th (before lockdown) to April 25th (after)

Author, Year, Country	Study design	Themes discussed
**Canevelli, 2020 (41) Italy**	Editorial	• General functional decline<br>• Negative impact on general well-being<br>• Negative impact on quality of life<br>• Feelings of loneliness/abandonment, triggering behavioral modifications in people with NCD following social distancing and isolation
**Wang, 2020 (44) China/UK**	Editorial	• Loneliness, abandonment, isolation and withdrawal
**Korczyn, 2020 (45) Israel**	Editorial	• Exacerbation of depression, anxiety, stress<br>• Induction of aggression
**Velayudhan, 2020 (46) UK**	Editorial	• Increase in anxiety, agitation, loneliness, depression<br>• Reduction in quality of life<br>• Boredom, inactivity, sedentary behavior<br>• Increase of cognitive impairment
**Dourado, 2020 (63) Brasil**	Editorial	• Disruption of social interactions may increase behavioral impairment in patients with dementia<br>• Increase in memory and orientation problems
**Gil, 2020 (36) Spain**	Commentary	• Relapse and exacerbation of NCD<br>• Face masks and physical distancing can disrupt facial familiarity and make it more difficult to recognize emotional facial expressions, which can provoke distress
**Barry, 2020 (2) UK**	Commentary	• Relapse and exacerbation of NCD
**Migliaccio, 2020 (38) France**	Commentary	• Increased isolation<br>• Difficulty to understand the situation<br>• Difficulty to conform to the restrictions in place
**Brown, 2020 (42) USA**	Commentary	• Feelings of loneliness/abandonment, behavioral modifications
**Howard, 2020 (26) UK**	Correspondence	• Increase in the proportion of dementia patients with antipsychotic prescriptions from March to July 2020<br>• Increase of the rate of dementia patients with antipsychotic prescriptions in March, April and May 2020 compared to the same months in 2019 and 2018

Table S2. Details of the retrieved studies in relation to the treatment impacts of COVID-19

Author, Year, Country	Study design	Methodology<br>i) Population<br>ii) Number of participants/groups<br>iii) Median/mean (SD) age<br>iv) Variables studied	Summary of results
**Capozzo, 2020 (29) Italy**	Longitudinal	i) Dementia patients receiving care at the Center for Neurodegenerative Disease and The Aging Brain of the University of Bari<br>ii) 32<br>iii) 66<br>iv) Satisfaction about telemedicine visits	• Most were consistently satisfied with the telemedicine visits<br>• 88% : satisfaction with the interview made by the neurologist<br>• 88% : willingness to continue to be placed in the telemedicine program
**Lai, 2020 (58) China**	Longitudinal	i) Community-dwelling people with cognitive impairment and their spousal caregivers from an activity day center<br>ii) 60 (30 : services through video conference, 30 : services through telephone only)<br>iii) Video conference : 72.87 (0.84), telephone : 72.73 (0.84)<br>iv) MoCA score, QoL-AD score	• Intervention with supplementary telehealth delivered via videoconferencing : resilience against a decline in general cognitive functioning
**Spaletta, 2020 (51) Italy**	Longitudinal	i) Patients with mild and major NCD from the outpatient memory clinic of the Santa Lucia Foundation IRCCS<br>ii) NA<br>iii) NA<br>iv) Comparison of scheduled vs conducted appointments at the clinic in 2019 vs 2020	• Decrease in appointments during March-April 2020 : 251 scheduled appointments canceled<br>• Increase in canceled appointments in March April 2020 vs 2019 : 70.4 % and 84.4% vs 17.3% and 27.3% for follow-up appointments ($p<0.001$), 76.2% and 57.1% vs 36.4% and 23.7% for first time appointments ($p=0.001$)<br>• 72% of patients missed their appointments during the lockdown period (66.7% first-time and 77.4% of follow-up)
**Chen, 2020 (52) China**	Longitudinal	i) Mental health patients<br>ii) NA<br>iii) NA<br>iv) Daily referrals to mental health service teams post lockdown	• Medium term effect : acceleration in referrals to mental health services teams after lockdown (+1.21 referrals/day)<br>• No acceleration in referrals for patients with dementia
**Barguilla, 2020 (32) Spain**	Longitudinal	i) Dementia and MCI patients from DegMar registry (Hospital del Mar)<br>ii) 60	• 16% of patients/families : difficulties in accessing medical resources

Author, Year, Country	Study design	Methodology<br>i) Population<br>ii) Number of participants/groups<br>iii) Median/mean (SD) age<br>iv) Variables studied	Summary of results
		iii) 75.4 ± 5.2<br>iv) Telephone questionnaire : assessment of medical care	• 33% of cases : provision of medical phone assistance<br>• 21% of patients : changes in psychopharmacological therapies during lockdown
**Padala, 2020 (47) USA**	Case report	i) Nursing home resident with Alzheimer's<br>ii) 1<br>iii) 81<br>iv) NA	• Kept in touch with his daughter through FaceTime, which calmed down the patient to his pre Covid level after a few days of facetime calls
**Giebel, 2020 (49) UK**	Cross sectional	i) Carers/people living with dementia from social care and social support services across the North West coast of England<br>ii) 50 (42 cares, 8 PLWD)<br>iii) Carers : 60 (8.8), PLWD : 63.6 (6.5)<br>iv) Theme qualitative analysis	• Significant reduction in social support service usage<br>- Causing loss of control, uncertainty, having to adapt to a new normal
**Giebel, 2020 (50) UK**	Cross sectional	i) PLWD and carers from the UK<br>ii) 569 (61 PLWD, 219 current carers; 66 former carers; 223 older adults)<br>iii) 67 (12)<br>iv) Short Warwick-Edinburgh Mental Well-Being Scale (SWEMWBS), Generalised Anxiety Disorder 7 (GAD-7), Personal Health Questionnaire 9 (PHQ-9)	• Weekly social support service usage and access to various services was significantly reduced with COVID-19<br>• Higher variations in social support service hours significantly predicted increased levels of anxiety in people with NCD
**Goodman-Casanov, 2020 (56) Spain**	Cross sectional	i) Community-dwelling older adults with mild cognitive impairment or mild dementia<br>ii) 93 (47 : intervention group with TV-AssistDem, 46 : TAU)<br>iii) 73.34 (6.07)<br>iv) Qualitative questionnaire about mental health and well being	• No significant differences in health and well-being between TV-AssistDem and TAU
**Benaque, 2020 (57) Spain**	Cross sectional	i) Patients with cognitive impairment from the ACE foundation<br>ii) NS<br>iii) NS	• After 1 week : drop of 60% of consultations<br>• Within 6 weeks : back to 78% of their regular activities

Author, Year, Country	Study design	Methodology<br>i) Population<br>ii) Number of participants/groups<br>iii) Median/mean (SD) age<br>iv) Variables studied	Summary of results
		iv) Evaluation of the number of consultations at the foundation after having switched from in person to online consultations	
**Van Maurik, 2020 (20)**	Cross sectional	i) Patients with dementia, MCI and subjective cognitive decline (SCD) and their caregivers<br>ii) 389 (SCD = 268, MCI = 35, dementia = 86)<br>iii) 67 ± 8<br>iv) Survey on psychosocial effects of Corona measures, including discontinuation of care	• 66% of patients (75% symptomatic and 61% cognitively normal) : report of discontinuation of care<br>• 36% symptomatic patients : discontinuation of day care, 32% : discontinuation of community care services<br>• 60% of patients were offered an alternative to day care → contact via phone<br>• 18% symptomatic patients : reported they needed more support than they were currently receiving<br>• 29% caregivers : reported they needed more support
**Cohen, 2020 (23) Argentina**	Cross sectional	v) Family members of patients at the Aging and Memory Center of FLENI with AD and related disorders living at home<br>vi) 119<br>vii) 81.16 ± 7.03 (patients)<br>v) Survey : change in rehabilitation services	• 76.9% : discontinuation of physical therapy during the pandemic<br>• 91.3% : discontinuation of occupational therapy<br>• 77.5% : discontinuation of cognitive rehabilitation
**Michalowsky, 2020 (53) Germany**	Cross sectional	i) Older population (>65 years old) in Germany who visited at least one of 1,095 GPs and internal specialists or 960 specialist practices between January and May 2020 or January and May 2019<br>ii) 2,447,356 patients<br>iii) 76.5 ± 8.4<br>viii) Diagnosis of incident dementia	• Reduction in diagnosis of incident dementia (-39%) in April and May 2020 compared to 2019
**Geddes, 2020 (59) Canada**	Review	i) Dementia patients<br>ii) NA<br>iii) NA<br>iv) Evaluation of remote cognitive assessment for dementia, logistical recommendations for	• Remote assessment = acceptable to patients/caregivers<br>• Primordial to consider informed consent, attention to privacy and to autonomy

Author, Year, Country	Study design	Methodology<br>i) Population<br>ii) Number of participants/groups<br>iii) Median/mean (SD) age<br>iv) Variables studied	Summary of results
		telemedicine services, framework for neurobehavioral status examination	• Neuropsychological tests administered by videoconferencing : good agreement with in-person assessment<br>• NCD neurological examination : reliably performed with telemedicine
**Sekhon, 2021 (60) Canada**	Review	i) Dementia patients in rural communities<br>ii) 12 articles included : cross sectional and longitudinal<br>iii) NA<br>iv) Evaluation of cognitive tests performed with telemedicine and of patient satisfaction with telemedicine	• General satisfaction with telemedicine<br>• Mixed results for the reliability of telemedicine in relation to adherence and cognitive tests
**Mok, 2020 (33) China**	Review	i) Dementia patients<br>ii) NA<br>iii) NA<br>iv) Challenges in the care and management of older people with dementia during COVID 19	• Disruption/reduction of community services and treatments because of confinement measures and redeployment of medical staff
**Simonetti, 2020 (34) United States**	Comprehensive review	i) Dementia patients<br>ii) 20 articles included<br>iii) NA<br>iv) NA	• Increase in pharmacological treatment strategies (antipsychotics and mood stabilizers)<br>• Shrinkage of community resources : limitation to the efficacity of non-pharmacological strategies<br>• Increased use of technology/electronic devices

Author, Year, Country	Study design	Themes discussed
**Cuffaro, 2020 (16) USA**	Editorial	• Challenges to telemedicine :<br>- Lack of availability of appropriate conditions to perform tele-consultations<br>- Knowledge/familiarity with modern technologies<br>- Ethic's concerns
**Shea, 2020 (43)**	Short editorial	• Increase of telemedicine for dementia patients

Author, Year, Country	Study design	Themes discussed
**Hong Kong**		
**Cheung, 2020 (61) New Zealand**	Editorial with case report	• Virtual adaptation of Cognitive Stimulation Therapy (CST)<br>• Successful transition of 10 in person CST groups to virtual CST
**Dai, 2020 (62) Hong Kong**	Editorial	• Benefits of telemedicine for dementia care<br>• Additional benefits from the sense of empowerment associated with the use of virtual technologies
**Dourado, 2020 (50) Brasil**	Editorial	• Restricted access to community services<br>• Non-attendance to consultations may worsen the cognition and overall functioning of dementia patients<br>• Telemedicine for dementia care is feasible, and allows the clinician to provide care in the social distancing context
**Rais, 2020 (64) Singapore**	Short editorial	• Suspension of dementia care services, leading to cognitive deconditioning<br>• Increase use of telemedicine<br>• Limitations to the use of telemedicine because of lack of access to technology, digital illiteracy and sensory impairment
**Canevelli, 2020 (41) Italy**	Editorial	• Benefits of telemedicine<br>- Reduced outpatient visits in crowded hospitals<br>- Minimized travel time to clinics<br>- Reduced waiting lists<br>• Similar efficacy to face-to-face meetings/activities
**Cheung, 2020 (61) New Zealand**	Editorial	• Successful implementation of therapy online instead of in person
**Barros, 2020 (55) Portugal**	Editorial	• Cancellation of group activities and care-related services for dementia patients<br>• Prohibition of visitors in nursing homes for 3 months<br>• Closure of day-care centers<br>• No formal support for dementia patients during the lockdown period
**Barry, 2020 (2) UK**	Commentary	• Challenges with medication administration and adherence<br>- Change in patient's routine<br>- Reduced caregiver input if he must self-isolate<br>- Reduced contact with general practitioner/community pharmacist<br>• Virtual modalities may not be adequate to perform physical and neurological examinations or some of the cognitive tests
**Killen, 2020**	Commentary	• Difficulties with telemedicine

Author, Year, Country	Study design	Themes discussed
**(37)**<br>**UK**		- Visual/language impairments in Lewis body dementia: struggles to interact virtually
**Sm-Rahman, 2020**<br>**(39)**<br>**Sweden**	Commentary	• Suspension of non-pharmacological interventions<br>• Increased isolation, lack of physical exercise, decreased social engagement, suspension of purposeful activity
**Tousi, 2020**<br>**(40)**<br>**USA**	Commentary	• Not all screening instruments to assess cognitive function are appropriate to be administered remotely : may affect assessment of cognitive impairment
**Brown, 2020**<br>**(42)**<br>**USA**	Commentary	• Suspension of non-pharmacological interventions, leading to isolation
**Ousset, 2020**<br>**(54)**<br>**France**	Commentary	• Suspension of outpatient visits and activities at a Memory clinic in France after lockdown<br>• Start of teleconsultations 1 week later via smartphones or tablets<br>• Drop in patients received at the Research Center : 52/week to 5/week
**Ibanez, 2020**<br>**(65)**<br>**Latin America**	Policy form	• Vulnerable and ethnic minorities suffering from NCD often receive inadequate care and will now be exposed to new constraints because of the pandemic
